# Hemodynamic comparisons of different shunt positions and geometrical model simplification strategies in the simulation of transjugular intrahepatic portosystemic shunt (TIPS)

**DOI:** 10.1038/s41598-024-82954-y

**Published:** 2024-12-28

**Authors:** Liu Yang, Yitao Zhang, Tianqi Wang

**Affiliations:** 1 School of Gongli Hospital Medical Technology, University of Shanghai for Science and Technology, No. 516 Jungong Road, Yangpu District, Shanghai, 200093 China; 2 School of Mechanical Engineering, University of Shanghai for Science and Technology, Shanghai, 200093 China

**Keywords:** Transjugular intrahepatic portosystemic shunt, Shunt position, Geometrical model simplification, Hemodynamics, Computational model, Computational models, Biomedical engineering

## Abstract

Transjugular intrahepatic portosystemic shunt (TIPS) is a widely used surgery for portal hypertensive patients, whose potential postoperative complications are closely related to the hemodynamic condition of the portal venous system. The selection of shunt position in the surgery may affect the postoperative hemodynamics; however, it is difficult for clinical studies to investigate the influence. Therefore, this study aims to employ the computational model simulating TIPS to compare the hemodynamic differences resulting from different shunt positions, and also to investigate the influences of different geometrical model simplification strategies used in the TIPS simulation. For this purpose, the clinical data of two representative patients were retrospectively collected, based on which, the computational hemodynamic models of the portal venous systems after TIPS were constructed, incorporating three typical shunt positions (i.e. shunt at the left/main/right portal vein) and three types of geometrical model simplification. Results showed that among the models with different shunt positions, the area-averaged flow velocity magnitudes in the shunts were very similar, while the model with shunt at the main portal vein showed the lowest postoperative portal pressure and the smallest area of high wall shear stress near the portal venous bifurcation. Among the models using different geometrical model simplification strategies, the simulated blood pressures at the main portal veins were very similar, but showed marked differences near the shunt inlets. Moreover, the area-averaged flow velocity magnitudes in the shunts were almost the same, while the velocity distributions differed a lot, leading to the different spatial distributions of wall shear stress near the portal venous bifurcations and shunt walls. These results on one hand suggested that placing the shunt at the main portal vein is more beneficial for the patient; on the other hand, they proved the feasibility of utilizing simplified model to save computational cost without losing the accuracy when the pressure at the main portal vein is mainly focused on. These findings would assist clinical decision-making and promote more accurate and efficient TIPS simulations.

## Introduction

Portal hypertension is a group of syndromes caused by a persistent increase in the portal pressure, mostly caused by liver cirrhosis. The typical symptoms include gastroesophageal variceal hemorrhage, refractory ascites, and hepatorenal syndrome which may be life-threatening without timely treatment^1,2^. To relieve portal hypertension especially ascites and gastroesophageal variceal hemorrhage, transjugular intrahepatic portosystemic shunt (TIPS) is currently an effective treatment option^3,4^. This minimally invasive surgery creates an artificial shunt in the liver parenchyma, which connects the portal vein and the hepatic vein. This shunt may lead some portal venous blood to flow directly into the inferior vena cava without traveling through the hepatic sinusoidal and therefore decrease the portal pressure^5,6^. Nevertheless, dozens of clinical studies have shown that TIPS is accompanied with many postoperative complications, such as insufficient pressure decrease, hepatic encephalopathy, and shunt stenosis^7^, which are closely related to the hemodynamic condition after shunt formation. In clinical practice, there are three available positions for the shunt formation, i.e. the left, main, and right portal veins, which are usually selected by the operator empirically^8,9^. Clinical studies found that the shunt position is associated with the risk of postoperative complications^10–12^, which is probably due to the different postoperative hemodynamics resulting from different shunt positions. Accordingly, elucidating the influence of shunt positions on postoperative hemodynamics is of great significance; however, due to the limitations of measurement devices and patient burden, it is difficult for clinical studies to investigate the influence.

In this context, computational hemodynamic modeling provides an alternative approach^13^. Previous studies employed computational models to address various issues related to the hepatic circulation. For example, Du et al. conducted the hemodynamic analysis of hepatic arteries for the early evaluation of hepatic fibrosis in biliary atresia^14^; Qu et al. investigated the mechanisms of the ascites volume differences between patients receiving a left or right hemi-liver graft liver transplantation using computational hemodynamic models. Recently, some studies constructed computational models to investigate the hemodynamic phenomena related to TIPS^15–18^. For example, Xiong et al. analyzed the hemodynamic effects of a streamlined controlled-expansion covered tapered stent for TIPS^15^; Jiang et al. constructed computational models of TIPS to quantify the influence of stent diameter and insertion position at the main portal vein^16^; Yin et al. built a series of models to investigate the influence of the entry site of the stent at the left or right portal vein and analyze the flow distribution owing to the shunt^17^. However, there has not been any study comparing the hemodynamic differences of shunt positions at the left, main, and right portal veins. Moreover, the geometrical model simplification strategies of the portal venous system used in previous studies were different where longer or shorter splenic vein and superior mesenteric vein were utilized in different studies. Owing to the quality of medical images or computational cost, geometrical model simplification is always implemented in the hemodynamic simulation, whose influence on the simulated hemodynamic variables in TIPS simulations is still not clear.

Therefore, this study aims to employ the computational model to simulate the hemodynamic variables after TIPS, compare the differences resulting from different shunt positions, and investigate the hemodynamic influences of different geometrical model simplification strategies used in the TIPS simulation. For this purpose, firstly, the clinical data of two representative patients were collected, based on which the preoperative geometrical models were reconstructed. Subsequently, the shunts at three typical positions were simulated and three different types of model simplification were conducted for each model, resulting 18 cases of geometrical models. Then, mesh models were generated and comparable boundary conditions were assigned based on in vivo measurements and empirical estimations. Finally, the simulated hemodynamic variables including blood pressure, flow velocity, and wall shear stress of multiple positions were quantitatively or qualitatively compared among the models with different shunt positions and different types of model simplification to clarify the influence of shunt positions and geometrical model simplification strategies on the postoperative hemodynamic variables.

## Methods

### Construction of geometrical models

The clinical data of two representative patients were collected from a previous study simulating the treatment of portal hypertension^19^, including the preoperative computed tomography angiography (CTA) images, and ultrasound-measured flow velocities in the main portal vein (MPV). The portal venous systems of the two patients both showed common structures and shapes, where Patient #1 had a relatively straight splenic vein (SV), while Patient #2 had a relatively curved SV.

**Fig. 1:**
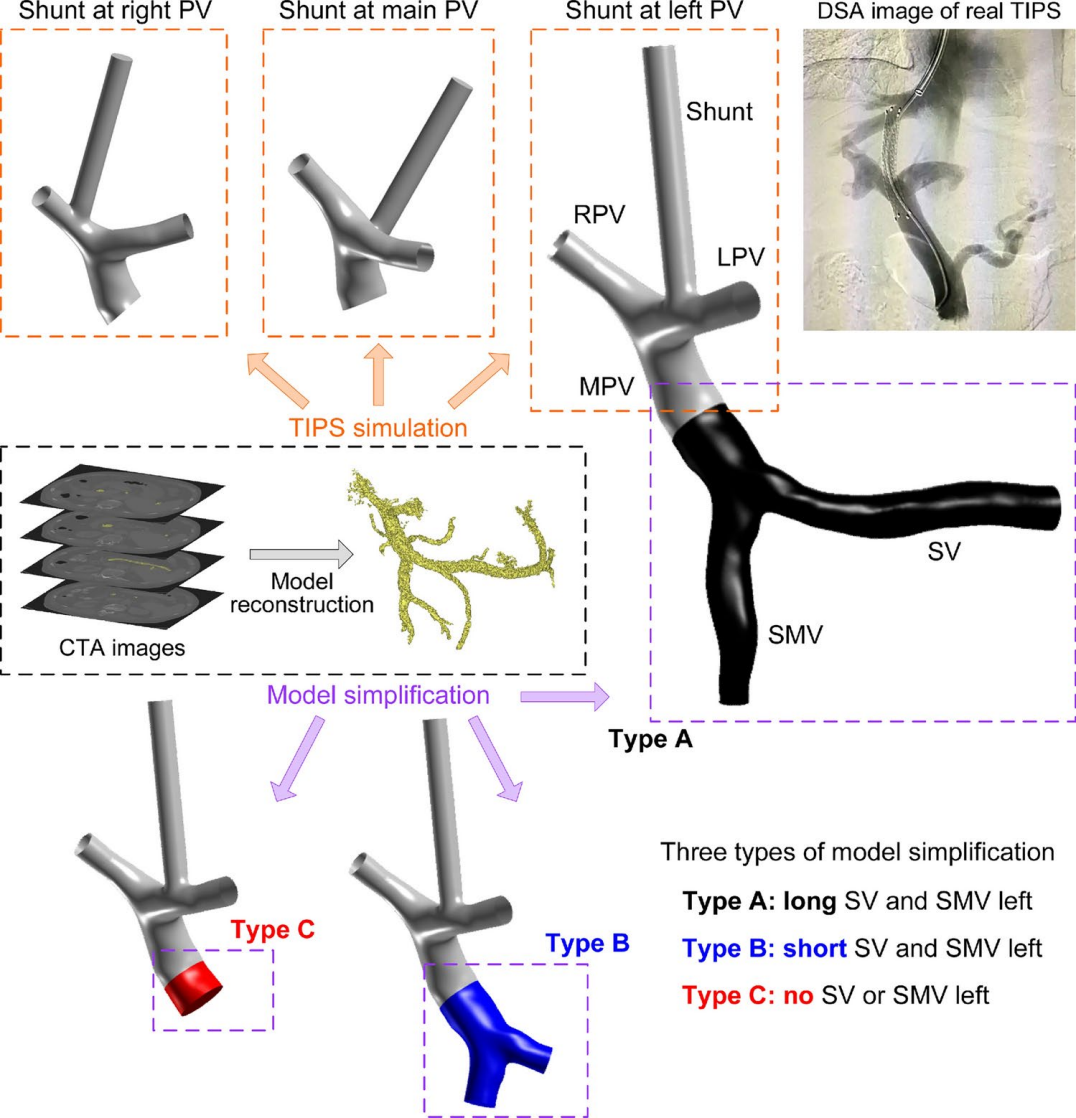
Geometrical models with three shunt positions and three types of model simplification (taking Patient #1 for example, so was Patient #2). The upper panels show the simulations of TIPS based on the preoperative geometrical model and real operation where the shunt was located at LPV, MPV, and RPV, respectively. The lower panels show three types of model simplification including Type A (in black), Type B (in blue), and Type C (in red). *LPV/MPV/RPV* left/main/right portal vein, *SV* splenic vein, *SMV* superior mesenteric vein. The digital subtraction angiography (DSA) image in the top right corner is a copy from public website.

The preoperative geometrical models were reconstructed from the CTA images by using the well-validated commercial package Mimics (Materialise, Belgium), as is shown in Fig. 1. Considering that 8 mm covered stent is usually inserted into the left portal vein (LPV), right portal vein (RPV), or MPV in clinical practice^20^, the simulation of TIPS for each preoperative model contained the construction of a 8 mm shunt with a uniform length of 60 mm in the aforementioned three typical positions, resulting three geometrical models for each of the two patients, as is shown in Fig. 1.

Each geometrical model was further simplified by removing the relatively smaller vessels of the portal venous system, leaving relatively larger ones including the SV, superior mesenteric vein (SMV), MPV, LPV, and RPV. This simplification was reasonable given that the smaller veins usually have lower blood flow whose influence on the simulated results is relatively smaller^21^. This simplified model with relatively intact long SV and SMV was named as Type A, whose simplification strategy was the same as that used in a previous study^16^. Considering that another previous study^17^ used the model of the portal venous system with shorter SV and SMV, the present study further involved another two types of model simplifications. Type B represented the model with relatively shorter SV and SMV, while Type C represented the model without SV or SMV, as is shown in Fig. 1. Finally, there were 18 cases of geometrical models (9 for Patient #1 and 9 for Patient #2) used for the simulations and comparisons.

### Generation of mesh models

**Fig. 2:**
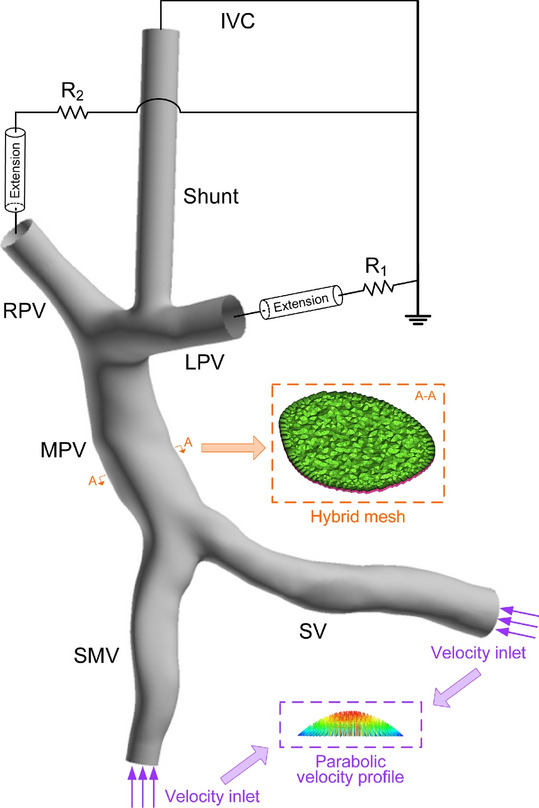
Mesh models and boundary conditions (taking Patient #1 for example, so was Patient #2). The mesh of a cross-section of MPV is shown in the orange dashed square. The inlets of SV and SMV are velocity inlets with parabolic velocity profiles. The outlets of LPV and RPV are connected with extensions and then connected with resistances before connected with the IVC. The outlet of the shunt is connected with the IVC directly. *LPV/MPV/RPV* left/main/right portal vein, *SV* splenic vein, *SMV* superior mesenteric vein, *IVC* inferior vena cava, *R* resistance.

Mesh models were generated by using ICEM CFD (ANSYS Inc., United States) with a hybrid meshing strategy, where the meshing of the entire fluid domain was firstly implemented using tetrahedral elements, followed by a mesh refinement treatment for the near-wall region by mapping 15 prism layers along the vascular walls, as is shown in Fig. 2. The minimum size of tetrahedral elements was set to 0.1 mm, and the thickness of the first near-wall prism layer was set to 0.02 mm, which was increased by a ratio of 1.1 toward the internal layers. The y+ of the prism layer was lower than 1, which guaranteed the correct solving when k–ω model was employed^22^. The grid convergence test performed on the model showed that further reducing the minimum size of the elements induced negligible changes in simulated hemodynamic variables (e.g. when the minimum size of the tetrahedral elements reduced from 0.1 to 0.05 mm, the relative variation of the portal pressure was only about 0.72%). Therefore, the aforementioned element sizes were applied to all models. Finally, each mesh model contained approximately 3 million (in Type C) to 6 million (in Type A) elements.

### Assignment of boundary conditions

For each patient, the preoperative volumetric flow rate in the MPV ($Q_{{{\text{MPV}}}}^{{{\text{pre}}}}$) was calculated based on the in vivo measured preoperative flow velocity in the MPV ($V_{{{\text{MPV}}}}^{{{\text{pre}}}}$) and the cross-sectional area of MPV ($A_{{{\text{MPV}}}}^{{{\text{pre}}}}$) derived from the geometrical model as Eq. (1):

1 $$Q_{{{\text{MPV}}}}^{{{\text{pre}}}} = V_{{{\text{MPV}}}}^{{{\text{pre}}}} \times A_{{{\text{MPV}}}}^{{{\text{pre}}}} .$$

On this basis, the postoperative volumetric flow rate in the MPV was estimated as two times of the preoperative one, according to the clinical measurements and the assignments in previous studies^17,23^. Subsequently, the volumetric flow rates in the SV ($Q_{{{\text{SV}}}}$) and SMV ($Q_{{{\text{SMV}}}}$) used in the computational models were assigned according to the Murray’s Law^24^, where the volumetric flow rate in each vessel was assumed to be proportional to the cube of the nominal diameter (SV diameter, *D*_SV_; SMV diameter, *D*_SMV_), as is shown in Eq. (2):

2 $$\left\{ {\begin{array}{*{20}l} {Q_{{{\text{SV}}}} = 2 \times Q_{{{\text{MPV}}}}^{{{\text{pre}}}} \times \frac{{D_{{{\text{SV}}}}^{3} }}{{D_{{{\text{SV}}}}^{3} + D_{{{\text{SMV}}}}^{3} }}} \hfill \\ {Q_{{{\text{SMV}}}} = 2 \times Q_{{{\text{MPV}}}}^{{{\text{pre}}}} \times \frac{{D_{{{\text{SMV}}}}^{3} }}{{D_{{{\text{SV}}}}^{3} + D_{{{\text{SMV}}}}^{3} }}} \hfill \\ \end{array} } \right..$$

The aforementioned diameters and flow rates are listed in Table 1. The flow rates in the SV and SMV or MPV were consistent in all the 9 models of each patient in order to make the simulated results comparable. Finally, mean flow velocities of the inlets could be calculated with the volumetric flow rates in combination with the corresponding cross-sectional area and were assumed to be constant considering the insignificant pulsation characteristics of the portal venous blood flow, which was in consistent with previous studies^25,26^. Parabolic velocity profiles were assigned to the inlets assuming the blood flow from upstream was fully developed Poiseuille flow^17,27^, as is shown in Fig. 2, which was a better choice than the flat velocity profile.

**Table 1:** Parameters of the geometrical models and boundary conditions.

Patient	#1			#2		
Parameter	*D* (mm)	*Q* (m^3^ s^−1^)	*R* (Pa s m^−3^)	*D* (mm)	*Q* (m^3^ s^−1^)	*R* (Pa s m^−3^)
MPV	16.45	7.86e−5	–	16.27	8.73e−5	–
SV	8.98	3.09e−5	–	14.18	3.55e−5	–
SMV	10.38	4.77e−5	–	16.08	5.18e−5	–
LPV	9.60	–	1.53e8	12.92	–	1.09e8
RPV	8.96	–	1.89e8	9.75	–	2.54e8

*MPV/LPV/RPV* main/left/right portal vein, *SV* splenic vein, *SMV* superior mesenteric vein, *D* diameter, *Q* volumetric flow rate, *R* flow resistance.

For the outlet LPV or RPV of each model, an extension were firstly connected followed by a resistance (*R*_1_ for LPV and *R*_2_ for RPV) which was further connected into the inferior vena cava (IVC) according to the anatomical structure^28–30^, as is shown in Fig. 2. Given the operation in real TIPS, the shunt outlet was connected directly to the IVC. Zero pressure was assumed to be the IVC pressure considering that the pressure gradient is usually focused on. The resistance (*R*_1_ or* R*_2_) was calculated by Eq. (3):

3 $$R_{i} = \frac{{P_{{{\text{pv}}}} }}{{Q_{{{\text{MPV}}}}^{{{\text{pre}}}} \times \frac{{D_{i}^{3} }}{{D_{1}^{3} + D_{2}^{3} }}}},\quad i = 1,2.$$

Here, *P*_pv_ was estimated as 25 mmHg which is the population-averaged portal pressure gradient of portal hypertensive patients before the surgery, since the in vivo measured data were missing; *D*_1_ and *D*_2_ represented the nominal diameters of the LPV and RPV, respectively. The calculation of *R*_1_ and *R*_2_ using Eq. (3) employed both the Murray’s Law and the relationship between blood pressure and volumetric flow rate^31–33^. The calculated *R*_1_ and *R*_2_ of the two patients are also listed in Table1. These assignments of the boundary conditions were similar with previous studies^16,17^ and were consistent in all the 9 models of each patient in order to make the simulated results comparable. Moreover, the vascular wall as well as the shunt wall of each model was assumed to be rigid, where no slip boundary condition was implemented, which was consistent with previous studies^16,17^.

### Calculation setting

The blood flow in the portal venous system is governed by the continuity and Navier–Stokes equations as is shown in Eq. (4) (Einstein summation convention applies to repeated indices):

4 $$\left\{ {\begin{array}{*{20}l} {\frac{{\partial u_{i} }}{{\partial x_{i} }} = 0} \hfill \\ {\frac{\partial }{{\partial x_{j} }}\left( {u_{i} u_{j} } \right) = - \frac{1}{\rho }\frac{\partial p}{{\partial x_{i} }} + \frac{\mu }{\rho }{\kern 1pt} \frac{{\partial^{2} u_{i} }}{{\partial x_{j} \partial x_{j} }}} \hfill \\ \end{array} } \right.,$$

where *u*_*i*_ is the blood flow velocity; *p* is the pressure; *ρ* (= 1060 kg/m^3^) and *μ* (= 0.0035 Pa s) are the density and dynamic viscosity of blood, respectively. Herein, the steady-state equation was used, given that the boundary conditions were constant and the simulated results would be convergent. According to previous studies^16^, flow velocity in the shunt is usually higher than the normal portal venous flow velocity remarkably. If the flow velocity in the 8 mm shunt was about 1.2 m/s (which was the simulated result of the present study and also the result reported in the previous study^16^), the Reynolds number would reach 2900 which is in the range of transition state of laminar flow and turbulent flow. Therefore, in the present study, the continuity and steady-state Reynolds-averaged Navier–Stokes (RANS) equations were actually solved as is shown in Eq. (5):

5 $$\left\{ {\begin{array}{*{20}l} {\frac{{\partial \overline{{u_{i} }} }}{{\partial x_{i} }} = 0} \hfill \\ {\overline{{u_{j} }} \frac{{\partial \overline{{u_{i} }} }}{{\partial x_{j} }} = - \frac{1}{\rho }\frac{{\partial \overline{p} }}{{\partial x_{i} }} + \frac{\mu }{\rho }{\kern 1pt} \frac{{\partial^{2} \overline{{u_{i} }} }}{{\partial x_{j} \partial x_{j} }} - \frac{{\partial \tau_{ij} }}{{\partial x_{j} }}} \hfill \\ \end{array} } \right.,$$

where the velocity and pressure in Eq. (4) are decomposed into mean and fluctuating parts: $u_{i} = \overline{{u_{i} }} + u^{\prime}_{i}$, $p = \overline{p} + p^{\prime}$, and $\tau_{ij} = \overline{{u^{\prime}_{i} u^{\prime}_{j} }}$ is the Reynolds-stress term that incorporates the effects of turbulent motions on the mean stresses^34^. This term requires additional modeling to fully resolve the system, which is fulfilled by the turbulence model. In the present study, k–ω model was employed to solve the RANS equations in the numerical simulations, which had already showed satisfactory performance in other hemodynamic studies^22^. The equations of blood flow together with the boundary conditions were numerically solved using Fluent (ANSYS Inc., United States), where 50,000 iterations or the residual lower than 1E−6 was set as the convergence criteria in each simulation. The parameters used in the k–ω model were the default values of the solver, which showed a good performance in solving the flow field.

### Data analysis

After the simulations of all the 18 cases, the hemodynamic variables of interest were extracted for the hemodynamic comparisons. In clinical practice, one of the main concerns of the surgeon is the postoperative portal pressure which is directly related to postoperative prognosis^35^. Besides, the shunt flow rate is also a noteworthy variable since postoperative hepatic encephalopathy is closely related to the excessive shunt flow^6,20^. Moreover, postoperative shunt stenosis is usually associated with thrombosis which may be associated with the biomechanical environment like abnormal wall shear stress (WSS) on the venous and shunt wall^19,26,27^. Therefore, in the present study, the simulated blood pressure, flow velocity, and wall shear stress were quantitatively and qualitatively compared among the models with different shunt positions and different geometrical model simplification strategies. As to WSS, the area of low wall shear stress (ALWSS) was calculated for each model with the estimated threshold value being 6 Pa for the portal venous bifurcation wall and 10 Pa for the shunt wall. Besides, the area of high wall shear stress (AHWSS) was also calculated for the portal venous bifurcation wall of the models with Type A simplification with the threshold value being 15 Pa. Considering that studies on the TIPS-related WSS are rare and thus there is no uniform threshold values for judging low and high WSS near the shunt, the aforementioned threshold values were all estimated according to the simulated spatial distribution of WSS, which was similar with the one used in the previous study^15^ and would ensure reasonable classifications. Moreover, the relative error (*E*_r_) of the ALWSS of the model with Type B or Type C simplification to that of the model with Type A simplification was also calculated for each patient with each shunt position as Eq. (6):

6 $$E_{{\text{r}}} = \frac{{{\text{ALWSS}} - {\text{ALWSS}}_{{\text{Type A}}} }}{{{\text{ALWSS}}_{{\text{Type A}}} }} \times 100\% .$$

## Results

### Comparisons of blood pressure of different models

**Fig. 3:**
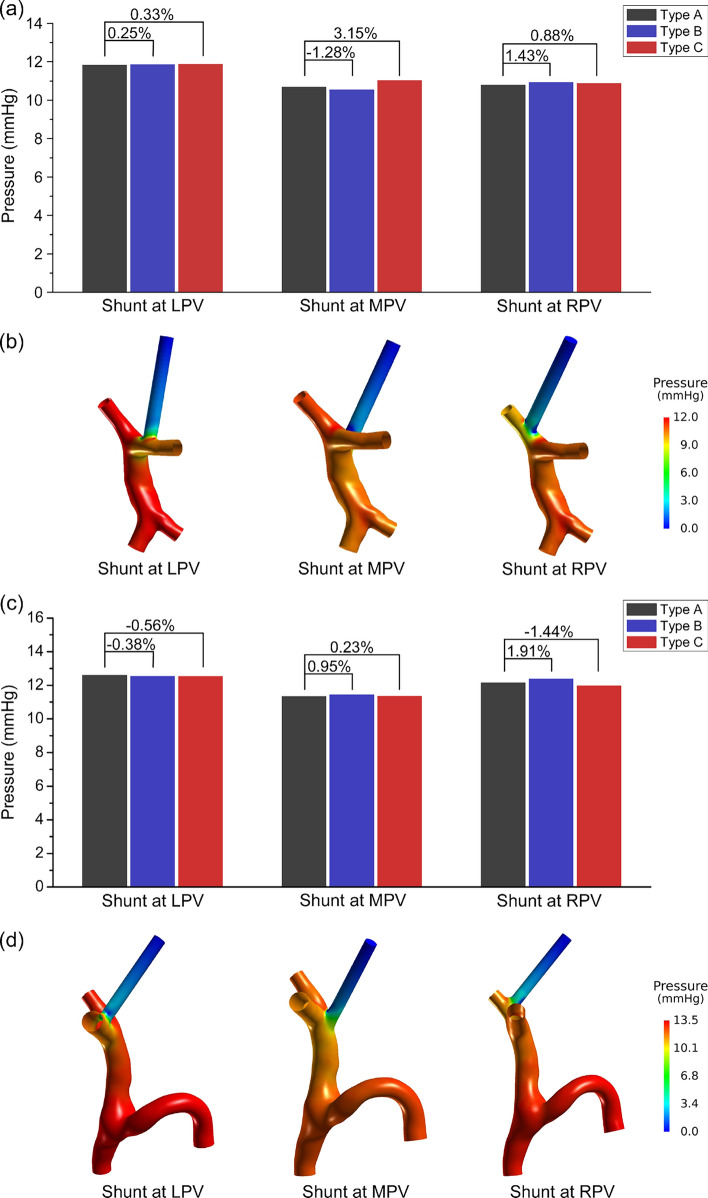
Comparisons of blood pressure of different models: (**a**) area-averaged pressure at the midsection of MPV of patient #1; (**b**) contours of pressure distribution of the model of Patient #1 with Type B simplification; (**c**) area-averaged pressure at the midsection of MPV of patient #2; (**d**) contours of pressure distribution of the model of Patient #2 with Type A simplification. *LPV/MPV/RPV* left/main/right portal vein.

**Fig. 4:**
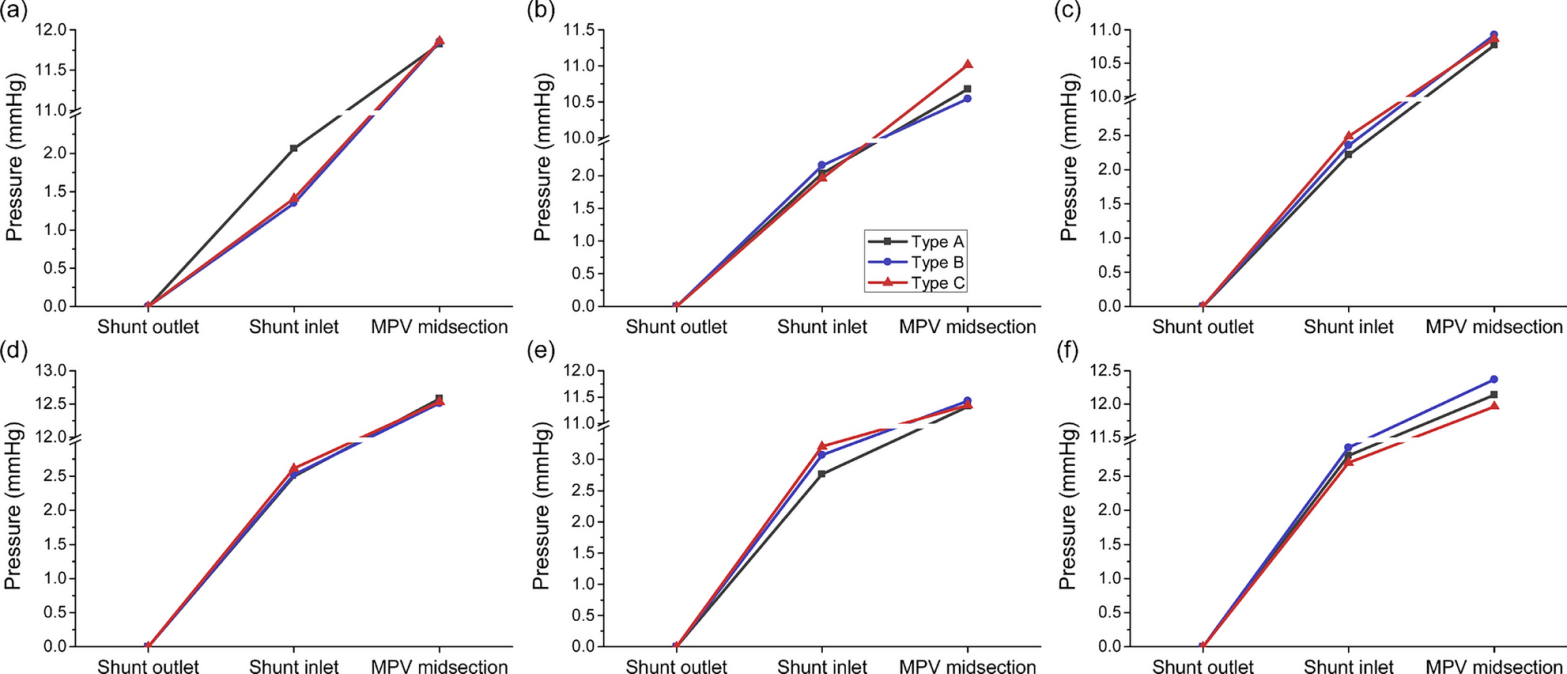
Comparisons of area-averaged blood pressure at shunt inlets and MPV midsections of the models with three types of model simplification: (**a**) Patient #1 with shunt at LPV; (**b**) Patient #1 with shunt at MPV; (**c**) Patient #1 with shunt at RPV; (**d**) Patient #2 with shunt at LPV; (**e**) Patient #2 with shunt at MPV; (**f**) Patient #2 with shunt at RPV. *LPV/MPV/RPV* left/main/right portal vein.

The simulated area-averaged pressures at the midsections of MPVs of the 9 cases of each patient were quantitatively compared in Fig. 3a or c. The relative error of the result of the model with Type B or Type C simplification to that of the model with Type A simplification was listed above the columns, which demonstrated that the maximum absolute value of the relative errors in Patient #1 and #2 were 3.15% and 1.91%, respectively. These results implied that the simulated pressure at the midsection of MPV was hardly influenced by the geometrical model simplification strategies. Moreover, the results also revealed that the simulated postoperative pressures in the models with the shunt at LPV and RPV showed minor difference, while placing the shunt at MPV resulted in the lowest postoperative portal pressure. The spatial distributions of pressure of the models with different shunt positions were shown in Fig. 3b and d, indicating that the pressure of the portal venous system showed intense variations near the portal venous bifurcation and the inlet of the shunt. Further quantitative comparisons of pressures at different positions illustrated that the drop of pressure reached about 10 mmHg between the midsection of MPV and the inlet of the shunt (50 mm far from the outlet with the total length of the shunt being 60 mm), while only about 2 mmHg between the inlet and outlet of the shunt, as is shown in Fig. 4. It is noted that in some cases, although the differences at the midsection of the MPV were small, the pressures at the inlet of the shunt differed remarkably among the models with different types of simplification, which implied that the geometrical model simplification strategies would influence the pressure near the portal venous bifurcation.

### Comparisons of flow velocities in different models

**Fig. 5:**
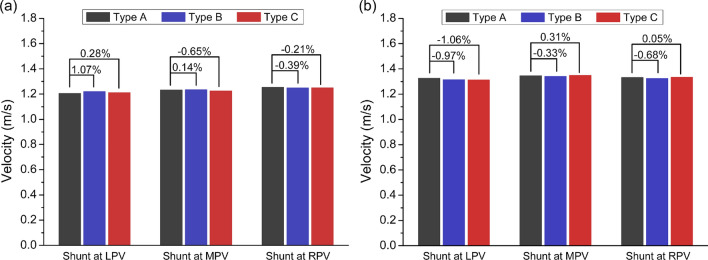
Comparisons of area-averaged flow velocity magnitudes at the shunt outlets of different models of Patient #1 (**a**), and Patient #2 (**b**). *LPV/MPV/RPV* left/main/right portal vein.

Figure 5 shows the comparisons of area-averaged flow velocity magnitudes at the shunt outlets of different models, illustrating the differences of the simulated volumetric flow rates in the shunts of the models with different shunt positions and geometrical model simplification strategies. The relative error of the result of the model with Type B or Type C simplification to that of the model with Type A simplification was listed above the columns, which demonstrated that the maximum absolute value of the relative errors in Patient #1 and #2 were both only about 1%. These results implied that the simulated volumetric flow rates in the shunts were almost the same whether which types of model simplification were used in the model constructions. Moreover, it can be observed that the shunt flows among the models with different shunt positions were also very similar in each patient.

Given that the real TIPS surgeries were not implemented for the patients in the present study, it was difficult to validate the present models based on patient-specific in vivo data. As an alternative, the simulated results of the present study were compared with the results reported in previous studies which had already been validated. As is shown in Table 2, the preoperative and postoperative portal pressure gradients and the blood flow velocity in the shunt were compared among the present study and several recent studies about TIPS^15–17^. Results demonstrated that the present simulated results were in the similar ranges with the data from literatures, illustrating the rationality of the simulated results using the present models.

**Table 2:** Comparisons of the simulated results with previous studies.

	Preoperative portal pressure gradient (mmHg)	Postoperative portal pressure gradient (mmHg)	Blood flow velocity in the shunt (m/s)
Approximate results of the present study	25	10–12	1.2–1.3
Xiong et al.^15^	21.04 ± 4.42	8.00 ± 2.70	About 1
Jiang et al.^16^	28–39	14.1–20.4	1.69–2.06
Yin et al.^17^	17–23	6.71–9.66	0.90–1.04

### Spatial distributions of wall shear stress

**Fig. 6:**
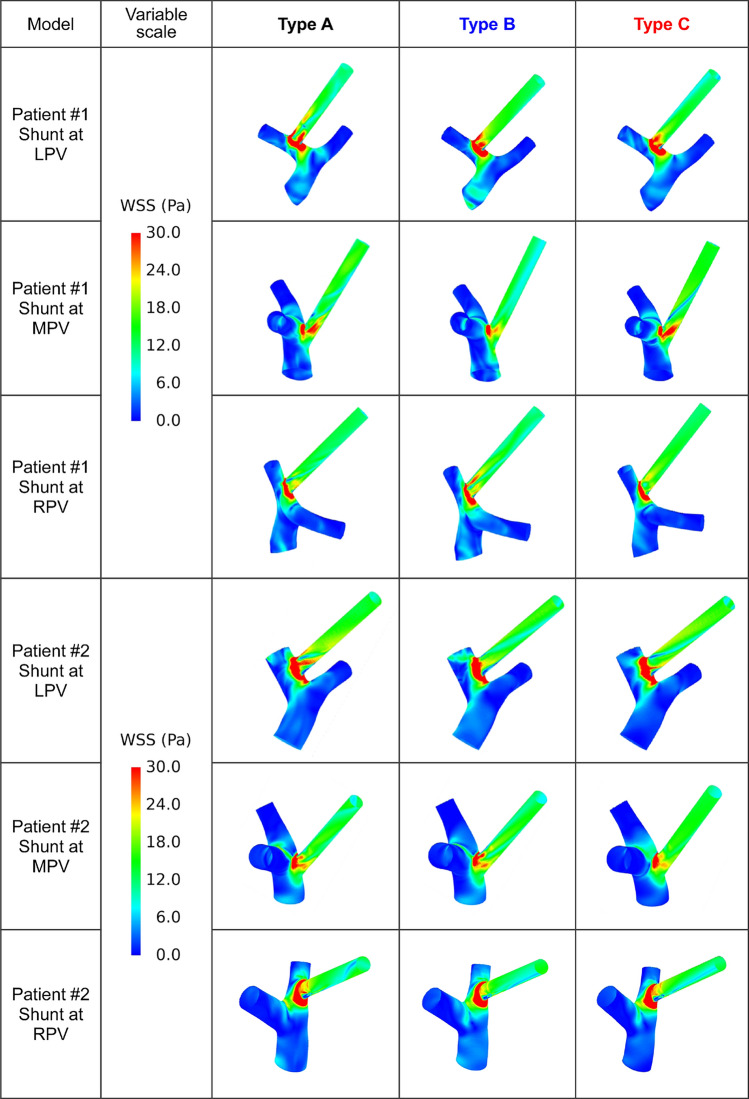
Space distributions of wall shear stress on the venous wall near the bifurcation of LPV and RPV and the wall of shunt of different models. *LPV/MPV/RPV* left/main/right portal vein, *WSS* wall shear stress.

The simulated spatial distributions of WSS on the venous wall near the portal venous bifurcation (i.e. the bifurcation of LPV and RPV) and the wall of shunt of the 18 cases are shown in Fig. 6. The differences among the models with Type A, Type B, and Type C simplification of each patient with each shunt position can be compared in each row qualitatively. Results showed that the distributions of low WSS on the venous walls were relatively regular in the models with Type C simplification, while relatively cluttered in the models with Type A or Type B simplification. Moreover, the distributions of high WSS near the inlets of the shunts showed remarkable differences in both the area and the shape of the regions. Further quantitative comparisons of the spatial distributions of WSS were evaluated by the relative error (*E*_r_) of ALWSS defined in “Data analysis” section, as is listed in Table 3. It can be found that the area of the regions with low WSS showed considerable difference. On the venous wall near the portal venous bifurcation, the maximum absolute value of *E*_r_ reached 10% in the model of Patient #1 with the shunt at RPV and Type B simplification, although most of the *E*_r_ was about 3%. As to the shunt wall, *E*_r_ was significantly higher, where the absolute value of *E*_r_ reached approximately 30% in some cases. These results indicated that the spatial distributions of WSS on the venous wall near the portal venous bifurcation and the wall of shunt were markedly influenced by the geometrical model simplification strategies. Furthermore, based on the relatively accurate simulated results (i.e. the results of the models with Type A simplification), it can be found that placing the shunt at the MPV induced the smallest area of high WSS near the portal venous bifurcation quantitatively and qualitatively (Table 4 and Fig. 6), indicating that the extreme value of WSS would be less if the shunt was at the MPV compared with the other two positions.

**Table 3:** Comparison of ALWSS among models with different types of simplification.

Patient	#1				#2			
Position	Bifurcation venous wall		Shunt wall		Bifurcation venous wall		Shunt wall	
Variable	ALWSS (cm^2^)	*E*_r_ (%)	ALWSS (cm^2^)	*E*_r_ (%)	ALWSS (cm^2^)	*E*_r_ (%)	ALWSS (cm^2^)	*E*_r_ (%)
Shunt at LPV								
Type A	24.38	–	3.58	–	24.76	–	1.70	–
Type B	22.85	− 6.28	2.72	− 24.02	24.82	0.24	1.92	12.94
Type C	24.38	0.00	3.25	− 9.22	25.26	2.02	1.98	16.47
Shunt at MPV								
Type A	27.28	–	3.53	–	26.17	–	3.13	–
Type B	26.00	− 4.69	3.86	9.35	26.77	2.29	3.08	− 1.60
Type C	26.78	− 1.83	2.45	− 30.59	25.69	− 1.83	2.08	− 33.55
Shunt at RPV								
Type A	25.35	–	2.86	–	25.93	–	2.02	–
Type B	22.80	− 10.06	3.36	17.48	25.80	− 0.50	2.02	0.00
Type C	25.16	− 0.74	2.24	− 21.68	26.23	1.16	1.43	− 29.21

*ALWSS* area of low wall shear stress, *E*_r_ relative error of ALWSS compared with Type A in each TIPS simulation.

**Table 4:** Comparison of AHWSS among models with different shunt positions.

AHWSS (cm^2^)	Patient	
Shunt position	#1	#2
Type A with shunt at LPV	3.687	3.092
Type A with shunt at MPV	2.337	2.405
Type A with shunt at RPV	2.898	3.182

*AHWSS* area of high wall shear stress.

### Streamlines and flow velocity distributions

**Fig. 7:**
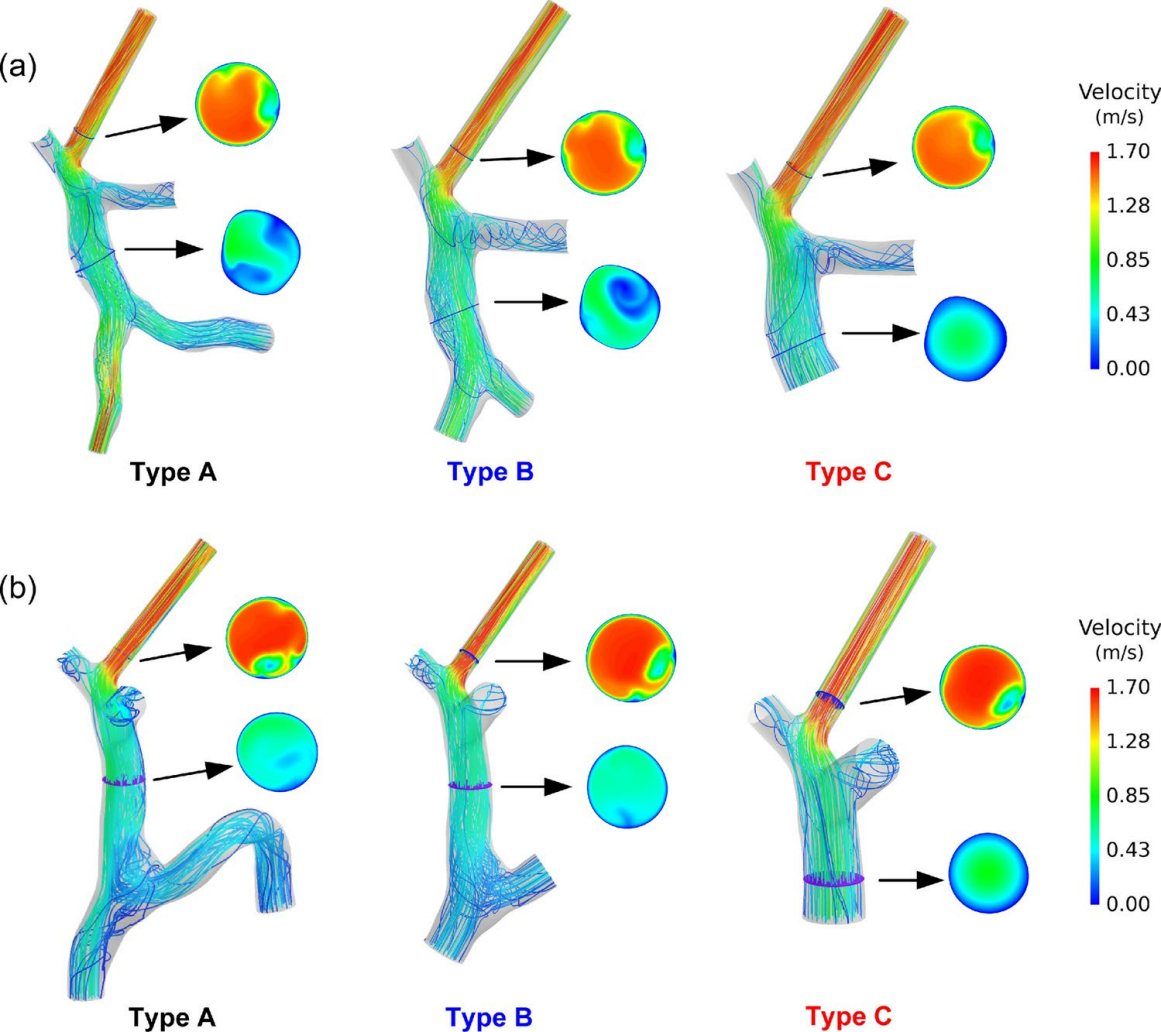
Comparisons of blood flow streamlines and contours of flow velocity magnitude in the midsections of MPV and shunt inlets of the models with three types of model simplification: (**a**) Patient #1 with shunt at RPV; (**b**) Patient #2 with shunt at RPV. *MPV/RPV* main/right portal vein.

Considering that the distribution of WSS is closely related to the flow pattern in the vessel and the local flow distribution is associated with the risk of thrombosis^25^, the details of flow distributions were also compared among the models with different types of model simplification. Figure 7 shows the comparisons of blood flow streamlines and contours of flow velocity magnitude in the midsections of MPV and shunt inlets. Although the area-averaged flow velocity magnitudes were almost the same in the shunts according to “Comparisons of flow velocities in different models” section, the spatial distributions of flow velocity differed a lot, which could partially explain the differences shown in the spatial distributions of WSS. It can be found that the flow velocity at the midsection of MPV of the model with Type C simplification was nearly the parabolic profile since the section was close to the inlet in this type, while in the models with Type A and Type B simplification, swirling flows existed and the distributions of flow velocity were distinct between the two types. Moreover, at the shunt inlets, the flow velocities were much higher and the distributions of flow velocity also showed significant differences, although the shunt inlets were relatively far from all the upstream inlets.

## Discussion

The postoperative complications of TIPS have been found associated with shunt positions selected for the surgery, while there is no clinical consensus claiming which position is a better choice^8,9^. Considering that the complications are closely related to the hemodynamic condition of the portal venous system, investigating the hemodynamic differences resulting from different shunt positions would assist clinical decision-making. This study employed the computational models with typical shunt positions to simulate the hemodynamic variables related to the postoperative complications, revealing that the shunt at LPV and RPV would result in similar postoperative portal pressures, while placing the shunt at the MPV would lead to lower results. Although the difference was only about 1–2 mmHg which seemed not very large, in clinical decision-making, sometimes if the portal pressure gradient is a little higher than the threshold value (12 mmHg, according to the clinical consensus^35^), placing the shunt at the MPV rather than LPV or RPV would make sense, which would lower the portal pressure and thus make the postoperative value meet the requirement of the guideline. Moreover, the area of extremely high WSS near the portal venous bifurcation would be smaller when the shunt was at the MPV rather than LPV or RPV (reported in “Spatial distributions of wall shear stress” section), which would lower the risk of postoperative shunt stenosis due to thrombosis, given that exposure to an abnormal biomechanical environment would result in endothelial dysfunction and thus increase the risk of thrombosis^27^. These results indicated that when a certain patient requires reducing more portal pressure or is more likely to suffer from thrombosis, the MPV may be a better choice for the shunt if the anatomical structure allows the operation. To the best of our knowledge, there are two groups also employing computational models to investigate the hemodynamics related to TIPS recently. One of the studies compared the hemodynamic influences of blind insertion into the MPV and different shunt diameters^16^. Another study compared the hemodynamic difference between the models with the shunts at LPV and RPV, mainly focusing on the flow distribution towards different sides^17^. In this context, the present study compared the postoperative hemodynamic differences of the surgeries with the shunts at LPV, MPV, and RPV, especially the hemodynamic variables related to postoperative complications (such as blood pressure, flow velocity, and WSS), which provided more useful insights into TIPS.

Moreover, given the quality of medical images or computational cost, the geometrical models reconstructed from medical images would always undergo model simplification, whose influence on TIPS simulations is still not clear^16,17^. Accordingly, this study also constructed the models with different geometrical model simplification strategies and compared the simulated hemodynamic variables. Results on one hand revealed that the simplification strategies are all acceptable when the blood pressure of MPV and the volumetric flow rate in the shunt are concerned; on the other hand demonstrated that the relatively intact geometrical model of the portal venous system (such as the model with Type A simplification in the present study) should be reserved when the hemodynamic variables (such as blood pressure, WSS, and the spatial distribution of flow velocity) near the portal venous bifurcation and the shunt inlet are mainly focused on. These findings demonstrated that the geometrical model simplifications strategies should be carefully selected in TIPS simulation, especially when the hemodynamic variables near the portal venous bifurcation are concerned. If the pressure of MPV or the volumetric flow rate in the shunt is the only concern without focusing on the local hemodynamic variables near the portal venous bifurcation, the simplified geometrical model like the model with Type B or even Type C simplification can be employed, which may output very similar results as the relatively intact model but has smaller sizes and thus consumes less computational cost. For example, in the present study, the model with Type A simplification consumed about 21 h, the model with Type B simplification consumed about 17 h, while the model with Type C simplification consumed only about 13 h under the same computer configuration saving about 38% computational cost. On the contrary, when the local hemodynamic variables like WSS and flow distributions are focused on (i.e. in the evaluation of postoperative shunt stenosis or risk of hepatic encephalopathy), the relatively intact geometrical model like the model with Type A simplification must be employed since the hemodynamic differences due to different geometrical model simplification strategies are remarkably large.

Admittedly, this study was subjected to certain limitations. The simulated results were lack of validation based on in vitro experiment. However, the comparisons of the present simulated results with the in vivo data from the literature validated the simulated results to some extent. Besides, the vascular wall was assumed to be rigid in the present study, which was not the same as real vessels in human body. Since the constant boundary conditions were assigned, the vascular wall would not vary significantly even if the deformable wall was used. As a result, it was reasonable that the influence of vascular elasticity was ignored. Moreover, only two patients were included in the present study. Nevertheless, the two patients were representative, which was reflected in both the population-averaged pathophysiological parameters and representative morphology. Specifically, one had relatively straight SV (Patient #1), while the other had relatively curved SV (Patient #2), which were the typical morphological features of the portal venous system in portal hypertensive patients^19^.

In summary, the present study constructed computational hemodynamic models of the portal venous systems based on the clinical data of two representative patients, incorporating three typical shunt positions and three types of geometrical model simplification to simulate TIPS. Results on one hand suggested that placing the shunt at the main portal vein is more beneficial for the patient; on the other hand, they proved the feasibility of utilizing simplified model to save computational cost without losing the accuracy when the pressure at the main portal vein is mainly focused on. These findings would assist clinical decision-making and promote more accurate and efficient TIPS simulations.

## Data Availability

The supporting data are available from the corresponding author upon request.

## References

[1] Ge, P. S. & Runyon, B. A. Treatment of patients with cirrhosis. *N. Engl. J. Med.* **375**, 767–777 (2016). 27557303 10.1056/NEJMra1504367

[2] Selicean, S. et al. Regression of portal hypertension: Underlying mechanisms and therapeutic strategies. *Hepatol. Int.* **15**, 36–50 (2021). 33544313 10.1007/s12072-021-10135-4 PMC7886770

[3] Berzigotti, A. Advances and challenges in cirrhosis and portal hypertension. *BMC Med.* **15**, 200 (2017). 29121925 10.1186/s12916-017-0966-6 PMC5680752

[4] Boyer, T. D. & Haskal, Z. J. The role of transjugular intrahepatic portosystemic shunt (TIPS) in the management of portal hypertension: Update 2009. *Hepatology* **51**, 306 (2010). 19902484 10.1002/hep.23383

[5] Vizzutti, F. et al. Transjugular intrahepatic portosystemic shunt (TIPS): Current indications and strategies to improve the outcomes. *Intern. Emerg. Med.* **15**, 37–48 (2020). 31919780 10.1007/s11739-019-02252-8

[6] Trebicka, J. et al. Smaller-diameter covered transjugular intrahepatic portosystemic shunt stents are associated with increased survival. *Clin. Gastroenterol. Hepatol.* **17**, 2793–2799 (2019). 30940552 10.1016/j.cgh.2019.03.042

[7] Tripathi, D. et al. Transjugular intrahepatic portosystemic stent-shunt in the management of portal hypertension. *Gut* **69**, 1173–1192 (2020). 32114503 10.1136/gutjnl-2019-320221 PMC7306985

[8] Miraglia, R. et al. Right vs left portal branch puncture in TIPS creation with controlled expansion covered stent: Comparison of hemodynamic and clinical outcomes. *Eur. Radiol.* **33**, 2647–2654 (2023). 36454260 10.1007/s00330-022-09280-7

[9] Zuo, K., Wang, C., Wang, J., Xia, F.-F. & Song, T. Transjugular intrahepatic portosystemic shunt through left branch versus right branch of portal vein: A meta-analysis. *Abdom. Radiol.* **46**, 1718–1725 (2021). 10.1007/s00261-020-02789-9 33009924

[10] Bai, M. et al. Shunting branch of portal vein and stent position predict survival after transjugular intrahepatic portosystemic shunt. *World J. Gastroenterol.* **20**, 774–785 (2014). 24574750 10.3748/wjg.v20.i3.774 PMC3921486

[11] Andring, B. et al. Effect of technical parameters on transjugular intrahepatic portosystemic shunts utilizing stent grafts. *World J. Gastroenterol.* **21**, 8110–8117 (2015). 26185383 10.3748/wjg.v21.i26.8110 PMC4499354

[12] Chen, S., Hu, P., Lin, Z. & Zhao, J. The effect of puncture sites of portal vein in TIPS with ePTFE-covered stents on postoperative long-term clinical efficacy. *Gastroenterol. Res. Pract.* **2019**, 2935498 (2019). 30728835 10.1155/2019/2935498 PMC6343182

[13] Formaggia, L., Quarteroni, A. & Veneziani, A. *Cardiovascular Mathematics: Modeling and Simulation of the Circulatory System* (Springer, 2009).

[14] Du, J. et al. Hemodynamic analysis of hepatic arteries for the early evaluation of hepatic fibrosis in biliary atresia. *Comput. Methods Programs Biomed.* **211**, 106400 (2021). 34551379 10.1016/j.cmpb.2021.106400

[15] Xiong, Z. et al. A streamlined controlled-expansion covered tapered stent for TIPS in the treatment of PHT. *J. Biomech.* **163**, 111937 (2024). 38246010 10.1016/j.jbiomech.2024.111937

[16] Jiang, L. et al. Do the stent blind insertion into the main portal vein (MPV) and stent diameter influence the surgical outcome of the transjugular intrahepatic portosystemic shunt (TIPS)?. *Comput. Biol. Med.* **164**, 107306 (2023). 37542920 10.1016/j.compbiomed.2023.107306

[17] Yin, K., Wang, X. & Zheng, T. Computational hemodynamic analysis for optimal stent position in the transjugular intrahepatic portosystemic shunt procedure. *J. Biomech.* **143**, 111303 (2022). 36126502 10.1016/j.jbiomech.2022.111303

[18] Ho, H. et al. Hemodynamic analysis for transjugular intrahepatic portosystemic shunt (TIPS) in the lver based on a CT-image. *IEEE Trans. Med. Imaging* **32**, 92–98 (2013). 23014713 10.1109/TMI.2012.2219882

[19] Wang, T., Yong, Y., Ge, X. & Wang, J. A computational model-based study on the feasibility of predicting post-splenectomy thrombosis using hemodynamic metrics. *Front. Bioeng. Biotechnol.* **11**, 1276999 (2024). 38274008 10.3389/fbioe.2023.1276999 PMC10808826

[20] Wang, Q. et al. Eight millimetre covered TIPS does not compromise shunt function but reduces hepatic encephalopathy in preventing variceal rebleeding. *J. Hepatol.* **67**, 508–516 (2017). 28506905 10.1016/j.jhep.2017.05.006

[21] Wang, T., Zhou, Z. & Liang, F. Influences of anatomorphological features of the portal venous system on postsplenectomy hemodynamic characteristics in patients with portal hypertension: A computational model-based study. *Front. Physiol.* **12**, 661030 (2021). 33912074 10.3389/fphys.2021.661030 PMC8072460

[22] Jodko, D., Obidowski, D., Reorowicz, P. & Jóźwik, K. Blood flows in end-to-end arteriovenous fistulas: Unsteady and steady state numerical investigations of three patient-specific cases. *Biocybern. Biomed. Eng.* **37**, 528–539 (2017).

[23] Stankovic, Z. et al. Effect of TIPS placement on portal and splanchnic arterial blood flow in 4-dimensional flow MRI. *Eur. Radiol.* **25**, 2634–2640 (2015). 25850890 10.1007/s00330-015-3663-x

[24] Murray, C. D. The physiological principle of minimum work. I. The vascular system and the cost of blood volume. *Proc. Natl. Acad. Sci. U.S.A.* **12**, 207–214 (1926). 16576980 10.1073/pnas.12.3.207 PMC1084489

[25] Yan, Y. et al. A novel potential mechanism for the development of portal vein thrombosis in cirrhosis based on portal hemodynamics. *Insights Imaging* **13**, 192 (2022). 36512292 10.1186/s13244-022-01330-4 PMC9748017

[26] Wei, W. et al. Wall shear stress in portal vein of cirrhotic patients with portal hypertension. *World J. Gastroenterol.* **23**, 3279–3286 (2017). 28566887 10.3748/wjg.v23.i18.3279 PMC5434433

[27] Wang, T., Liang, F., Song, G., Guan, J. & Zhou, Z. Predicting the risk of postsplenectomy thrombosis in patients with portal hypertension using computational hemodynamics models: A proof-of-concept study. *Clin. Biomech.* **98**, 105717 (2022). 10.1016/j.clinbiomech.2022.105717 35834965

[28] Wang, T. et al. A computational model-based study on the exchangeability of hepatic venous pressure gradients measured in multiple hepatic veins. *Med. Eng. Phys.* **84**, 28–35 (2020). 32977920 10.1016/j.medengphy.2020.07.022

[29] Wang, T., Liang, F., Zhou, Z. & Qi, X. Global sensitivity analysis of hepatic venous pressure gradient (HVPG) measurement with a stochastic computational model of the hepatic circulation. *Comput. Biol. Med.* **97**, 124–136 (2018). 29723809 10.1016/j.compbiomed.2018.04.017

[30] Wang, T., Liang, F., Zhou, Z. & Shi, L. A computational model of the hepatic circulation applied to analyze the sensitivity of hepatic venous pressure gradient (HVPG) in liver cirrhosis. *J. Biomech.* **65**, 23–31 (2017). 29042056 10.1016/j.jbiomech.2017.09.023

[31] Moriyasu, F. et al. Measurement of portal vascular resistance in patients with portal hypertension. *Gastroenterology* **90**, 710–717 (1986). 2935445 10.1016/0016-5085(86)91127-3

[32] Alastruey, J., Parker, K. H., Peiró, J. & Sherwin, S. J. Lumped parameter outflow models for 1-D blood flow simulations: Effect on pulse waves and parameter estimation. *Commun. Comput. Phys.* **4**, 317–336 (2008).

[33] Shi, Y., Lawford, P. & Hose, R. Review of zero-D and 1-D models of blood flow in the cardiovascular system. *BioMed. Eng. OnLine* **10**, 33 (2011). 21521508 10.1186/1475-925X-10-33 PMC3103466

[34] Alfonsi, G. Reynolds-averaged Navier–Stokes equations for turbulence modeling. *Appl. Mech. Rev.* **62**, 040802 (2009).

[35] De Franchis, R. et al. Baveno VII—Renewing consensus in portal hypertension. *J. Hepatol.* **76**, 959–974 (2022). 35120736 10.1016/j.jhep.2021.12.022 PMC11090185

